# ms-data-core-api: an open-source, metadata-oriented library for computational proteomics

**DOI:** 10.1093/bioinformatics/btv250

**Published:** 2015-04-24

**Authors:** Yasset Perez-Riverol, Julian Uszkoreit, Aniel Sanchez, Tobias Ternent, Noemi del Toro, Henning Hermjakob, Juan Antonio Vizcaíno, Rui Wang

**Affiliations:** ^1^European Molecular Biology Laboratory, European Bioinformatics Institute (EMBL-EBI), Wellcome Trust Genome Campus, Hinxton, Cambridge, CB10 1SD, UK,; ^2^Ruhr-Universität Bochum, Medizinisches Proteom-Zenter, Medical Bioinformatics, ZKF, E.142, Universitätsstr. 150, D-44801 Bochum, Germany and; ^3^Department of Proteomics, Center for Genetic Engineering and Biotechnology, Ciudad de la Habana, Cuba

## Abstract

**Summary:** The ms-data-core-api is a free, open-source library for developing computational proteomics tools and pipelines. The Application Programming Interface, written in Java, enables rapid tool creation by providing a robust, pluggable programming interface and common data model. The data model is based on controlled vocabularies/ontologies and captures the whole range of data types included in common proteomics experimental workflows, going from spectra to peptide/protein identifications to quantitative results. The library contains readers for three of the most used Proteomics Standards Initiative standard file formats: mzML, mzIdentML, and mzTab. In addition to mzML, it also supports other common mass spectra data formats: dta, ms2, mgf, pkl, apl (text-based), mzXML and mzData (XML-based). Also, it can be used to read PRIDE XML, the original format used by the PRIDE database, one of the world-leading proteomics resources. Finally, we present a set of algorithms and tools whose implementation illustrates the simplicity of developing applications using the library.

**Availability and implementation:** The software is freely available at https://github.com/PRIDE-Utilities/ms-data-core-api.

**Supplementary information:** Supplementary data are available at *Bioinformatics* online

**Contact:** juan@ebi.ac.uk

## 1 Introduction

The Proteomics Standards Initiative (PSI) has developed and actively promotes the use of open standard data formats to represent the data produced in mass spectrometry (MS) based proteomics experiments (including technical and biological metadata). Three of the most broadly used formats are: mzML (Martens *et al*., 2011) to capture the ‘primary’ data (the spectra and chromatograms), mzIdentML (Jones *et al*., 2012) to report peptide identifications as well as the inferred protein identifications, and the tab-delimited mzTab format (Griss *et al*., 2014) that can represent both identification and quantification results. There is increasing interest in new software tools and libraries that can work with these standards. As a result, a set of software libraries in different programming languages has been created (Perez-Riverol *et al*., 2014). However, having these independent libraries for different formats can complicate the development of new software. Developers typically have to invest considerable time and effort in basic functionality such as converting data structures between formats, shifting their focus away from the novel aspects of their software. In parallel, the volume of MS proteomics data available in the public domain keeps growing. This presents immense potential for quality assessment and data reanalysis (Perez-Riverol *et al*., 2015). A large proportion of the public data is in standard formats, which are heavily promoted by the resources part of ProteomeXchange (Vizcaino *et al*., 2014).

Here we present the ms-data-core-api, an open-source Java Application Programming Interface (API) to efficiently handle the main data types in MS proteomics workflows, ranging from spectra to peptide/protein identifications to quantitative results. The current version supports three major PSI data standards (mzML, mzIdentML and mzTab) and the majority of mass spectra file formats (mzXML, mzData, mgf, pkl, dta, ms2, apl). This makes ms-data-core-api the first open-source API supporting both identification and quantitation PSI file formats. In addition, as a key feature, it fully supports access to data stored in the PRIDE database (Vizcaino *et al*., 2013) by also supporting the PRIDE XML format, providing access to all the projects available in this older format. We also introduce a rapidly growing set of algorithms and tools whose implementation helps to illustrate the simplicity of developing applications based on ms-data-core-api.

## 2 Design and implementation

**Fig. 1.**
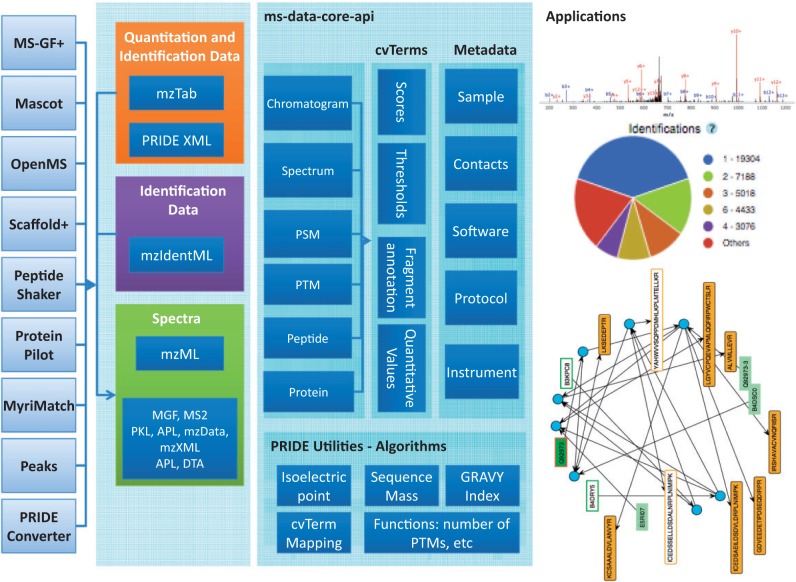
Overview of the design of the ms-data-core-api

The ms-data-core-api library provides a unified access interface to different proteomics MS-derived data types, independent of the format-specific details (Fig. 1). This interface provides methods to access and retrieve information on metadata, chromatograms, spectra, peptide spectrum matches (PSMs), peptides, proteins, protein modifications including post-translational modifications (PTMs) and quantitative results (Supplementary information, section S1.2). The biggest advantage of using the library is that any application based on it is largely file format agnostic. Following a modular design, many independent libraries were grouped at the same dependency level (Supplementary information, Fig. S1). The developed data model provides adapters that can translate the input data from the different source files into the core data structures, enabling the support of widely used formats (Supplementary information, Table S1). Crucially, the output from some of the most used analysis software in the field supports or can be converted to these highly popular formats, and then supported by the library (Fig. 1).

The API is composed of four functional components: (i) the data models incorporating all data structures (chromatograms, spectra, peptide/protein identifications, and quantitative information; Supplementary information, section S1.2.2); (ii) the transformers between the native file data representation and the data model; (iii) the cache system warranting fast and efficient access to the data (Supplementary information 1, section S1.2.3); and (iv) the data access controllers that can interface with external tools and libraries.

*Metadata-driven design*: The data model has three major components: proteomics data, features and properties and metadata (Fig. 1). The proteomics data comprise the spectra related information (intensities and masses), peptide/protein identifications (sequences, protein identifiers) and quantitation information. Also, the proteomics data model encodes the associated features (e.g. scores, thresholds, etc) as *cvParams*, which refer to either a controlled vocabulary (CV) or an ontology (e.g. the PSI-MS CV), and *userParams,* which are user-defined parameters to represent information not yet included in CVs and ontologies. The API also contains general metadata on the experimental set-up. For example, metadata on the protein identification protocol such as software, enzyme and search database are part of the *ProteinDetectionProtocol* class.

*Cache design and PRIDE utilities*: The design of the ms-data-core-api aims to achieve an optimal balance between memory consumption and access performance to the data by using a custom caching implementation (Supplementary information, Fig. S3). Most of the data structures in the API are cached as key-value entries depending on access patterns. Data objects are cached as a whole if they are requested frequently, whereas objects requested less often are cached as mappings to their locations within the source file for fast random access. Also, new refinements were introduced in the file format native readers (Supplementary information, Section S1.2.2). The independent PRIDE Utilities module (Fig. 1) provides a *cvParam* mapper that enables ms-data-core-api to homogenize *terms* across different file formats. It also includes functions to predict isoelectric point (Perez-Riverol *et al*., 2012), monoisotopic mass, and the GRAVY index (Supplementary information, Section S1.2.4). As shown in the code snippet below, the calculation of these properties for all the supported formats takes minimum effort:

MzIdentMLControllerImpl controller = new MzIdentMLControllerImpl(new File(“file.mzid”));
Collection<Comparable> proteins = controller.getProteinIds();
for(Comparable id: proteins)
 for(Comparable pepId: controller.getPeptideIds(id))
  double pI=                    IsoelectricPointUtils.calculate(controller.getPeptideSequence(id,pepId)));

*Exporting to mzTab*: The library includes a range of options to export the core data models and the processed results to mzTab files. The current version enables the conversion from mzIdentML and PRIDE XML to mzTab files including a set of filters to select only the high-quality peptide and protein identifications (Supplementary information, Section S1.2.4). As shown in the code snippet below, the conversion of mzIdentML to mzTab takes minimum effort:

MzIdentMLControllerImpl controller = new MzIdentMLControllerImpl(new File(“input.mzid”));
AbstractMzTabConverter mzTabconverter = new MzIdentMLMzTabConverter(controller);
MZTabFile mzTabFile = mzTabconverter.getMZTabFile();
MZTabFileConverter checker = new MZTabFileConverter();
checker.check(mzTabFile);
mzTabFile.printMZTab(new FileOutputStream(“output.mztab”));

## 3 Tools and future directions

Various algorithms, tools and pipelines have already been developed using the ms-data-core-api including PRIDE Inspector, the PRIDE internal submission pipeline, HI-bone and the PIA protein inference algorithm, among others (Perez-Riverol *et al*., 2013; Vizcaino *et al*., 2013; Wang *et al*., 2012; Supplementary information, Section S3). The widespread use of the library ensures its stability, continued development, and community support. The ms-data-core-api library is freely available, and is released under the Apache 2.0 license at https://github.com/PRIDE-Utilities/ms-data-core-api.

## Funding

Y.P-R. is supported by the BBSRC ‘PROCESS’ grant [BB/K01997X/1]; R.W. by the BBSRC ‘Quantitative Proteomics’ grant [BB/I00095X/1]; T.T. by the BBSRC ‘Proteogenomics’ grant [BB/L024225/1]; J.A.V. and N.d.T. by the Wellcome Trust [grant number WT101477MA] and J. U. by PURE (Protein Unit for Research in Europe), a project of North Rhine-Westphalia, Germany.

*Conflict of Interest*: none declared.

## Supplementary Material

Supplementary Data
