# Supplementary material for: ms-data-core-api: an open-source, metadata-oriented library for computational proteomics
Source: Bioinformatics. 2015 Apr 24;31(17):2903–5. doi: 10.1093/bioinformatics/btv250 (PMC4547611; doi:10.1093/bioinformatics/btv250)
Supplement: Supplementary Data [file supp_31_17_2903__index.html]

ms-data-core-api: An open-source, metadata-oriented library for computational proteomics — ms-data-core-api: an open-source, metadata-oriented library for computational proteomics — ms-data-core-api: an open-source, metadata-oriented library for computational proteomics — Supplementary Data 

# ms-data-core-api: an open-source, metadata-oriented library for computational proteomics

## Supplementary Data

files

**Files in this Data Supplement:**

- Supplementary Data - pdf file
