# Supplementary material for: ms-data-core-api: an open-source, metadata-oriented library for computational proteomics
Source: Bioinformatics. 2015 Apr 24;31(17):2903–5. doi: 10.1093/bioinformatics/btv250 (PMC4547611; doi:10.1093/bioinformatics/btv250)
Supplement: Supplementary Data [file supp_btv250_Supplementary_Information_final.pdf]

Application Note

ms-data-core-api: An open-source, metadata-oriented library for computational proteomics

Yasset Perez-Riverol [a], Julian Uszkoreit [b], Aniel Sanchez [c], Tobias Ternent [a], Noemi del Toro [a], Henning Hermjakob [a], Juan Antonio Vizcaíno [a,*] & Rui Wang [a]

[a] European Molecular Biology Laboratory, European Bioinformatics Institute (EMBL-EBI), Wellcome Trust Genome Campus, Hinxton, Cambridge, CB10 1SD, UK.
[b] Ruhr-Universität Bochum, Medizinisches Proteom-Zenter, Medical Bioinformatics, ZKF, E.142, Universitätsstr. 150, D-44801 Bochum, Germany.
[c] Department of Proteomics, Center for Genetic Engineering and Biotechnology. Ciudad de la Habana. Cuba.

Summary: The ms-data-core-api is a free, open-source library for developing computational proteomics tools and pipelines. The Application Program Interface, written in Java, enables rapid tool creation by providing a robust, pluggable programming interface and common data model. The data model is based on controlled vocabularies/ontologies and captures the whole range of data types included in common proteomics experimental workflows, going from spectra to identifications to quantitative results. The library contains readers for three of the most used Proteomics Standards Initiative standard file formats: mzML, mzIdentML, and mzTab. In addition to mzML, it also supports other common mass spectra formats: dta, ms2, mgf, pkl, apl (text-based), mzXML and mzData (XML-based). Also, it can be used to read PRIDE XML, the original format used by the PRIDE database, one of the world-leading proteomics resources. Finally, we present a set of algorithms and tools whose implementation illustrates the simplicity of developing applications using the library.

Supplementary Information Document Contents

1. ms-data-core-api Technical Implementation 3
   1.1. General Information 3
   1.2 Design and Implementation (ms-data-core-api) 3
   1.2.1 Data Model 5
   1.2.2 Cache Design 7
   1.2.3 PRIDE Utilities: Computational proteomics functionalities 9
   1.2.4 Exporting to mzTab 11

2. Updated ms-data-core-api dependencies 13
   2.1 PRIDE XML JAXB 13
   2.2 jmzML 13
   2.3 jmzIdentML 14
   2.4 jmzReader 15

3. Algorithms and tools built on top of the ms-data-core-api 16
   3.1 PRIDE Inspector Toolsuite 16
   3.2 PRIDE submission pipeline 16
   3.3 HI-bone 17
   3.4 Protein Inference Algorithms (PIA) 17

4. Coding examples 19

5. References 22

1. ms-data-core-api Technical Implementation

1.1. General Information

System Requirements:

- Java: JRE 1.6 +
- CPU: 2 gigahertz (GHz) or faster 32-bit or 64-bit processor
- Memory: 2 gigabyte (GB) RAM
- Hard Disk: 50 MB available
- Platform: It has been tested on Mac OS X, Linux and Windows

Website of the project:

https://pride-utilities.github.io

Java Documentation (javadoc):

https://pride-utilities.github.io/ms-data-core-api/javaDoc

Wiki Page:

https://github.com/PRIDE-Utilities/ms-data-core-api/wiki

Source Code:

https://github.com/PRIDE-Utilities/ms-data-core-api

Issues:

https://github.com/PRIDE-Utilities/ms-data-core-api/issues

1.2 Design and Implementation (ms-data-core-api)

The data object module is an abstraction layer between the data and the data representation (Figure 1). It is implemented using plain Java objects, which are core objects used to handle information across different input formats. In fact, different formats often don't have a unified way of representing the same information, and they may also contain different aspects of experimental details. For instance, spectrum related metadata in the mzML format (Martens, et al., 2011) and PRIDE XML are formatted differently, and the peptide and protein identifications in PRIDE XML, mzIdentML (Jones, et al., 2012) and mzTab formats (Griss, et al., 2014) are represented also in different ways. The data model represents a standardized view of the information in the underlying data sources. While reading, the raw content

from the data source is first converted into objects of the data module. This process of data transformation naturally depends on the input data source and a utility API (Application Programming Interface) is provided to facilitate the extraction of the information from the original data source.

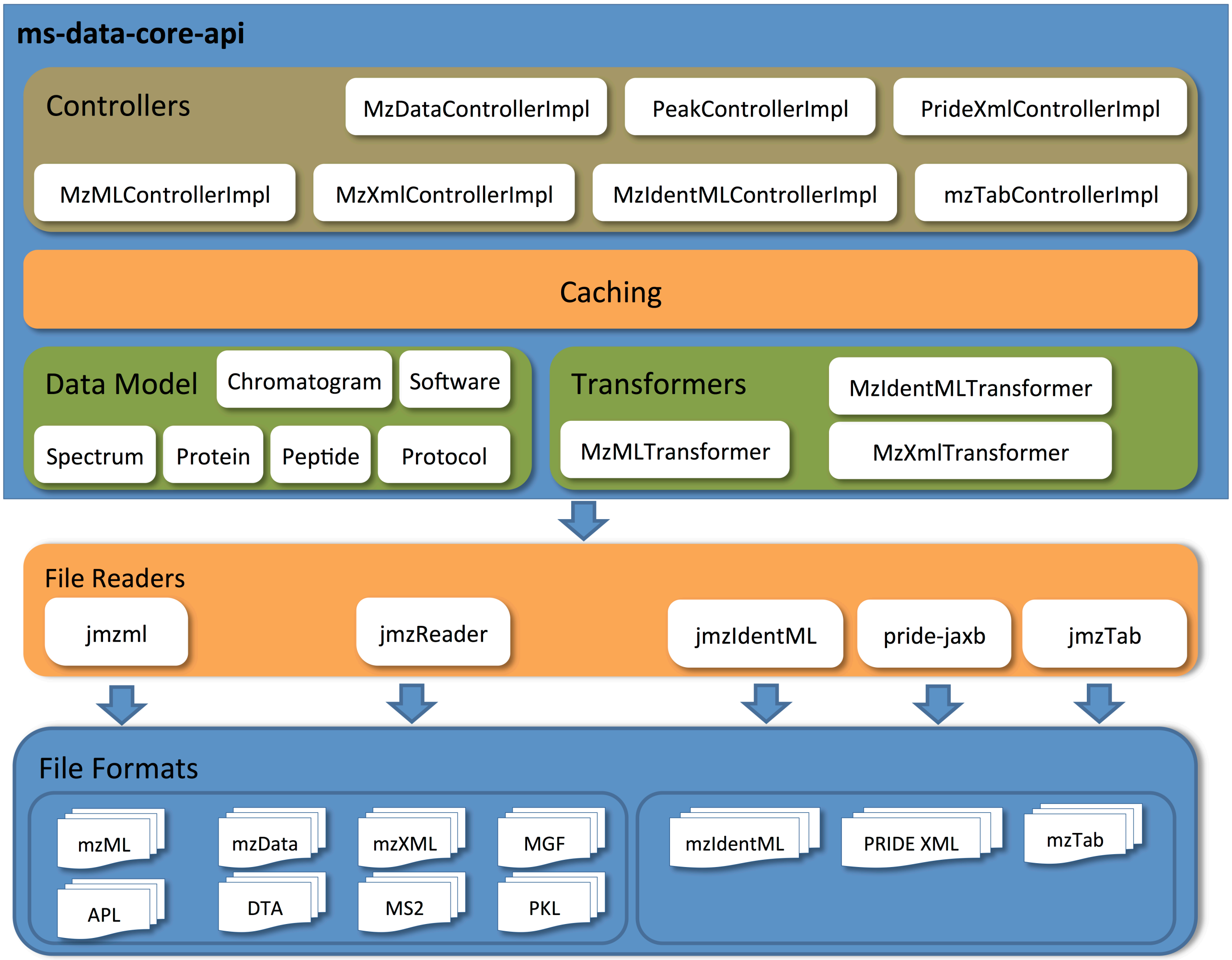

Figure 1: Data Object Model

The current Data Object Model supports file formats containing spectrum, identification and quantitation information (Table 1). Specifically, it supports three major PSI (Proteomics Standards Initiative) data standard formats: mzML, mzIdentML and mzTab. Every file format is read using different file-specific readers and translated using Transformers to the Data Object Model. The Data Object Model consists of different classes representing the main data types in proteomics studies such as chromatogram, spectrum, peptide, protein, etc. A novel cache system was implemented in order to increase the performance and memory usage of the library. This cache system is especially useful for GUI (Graphical User Interface) components that require concurrent operations in the same data. Finally, a set of controllers that extends a general *DataAccessController* Interface enables the data retrieval from the Data Object Model.

	File Format	Used API
Spectra File Formats	mzML	jmzML(Cote, et al., 2010)
	mzXML	jmzReader(Griss, et al., 2012)
	mzData	jmzReader(Griss, et al., 2012)
	Peak files (mgf, ms2, dta, pkl, apl)	jmzReader(Griss, et al., 2012)
Identification File Formats	PRIDE XML	pride-jaxb
	mzIdentML	jmzIdentML(Reisinger, et al., 2012)
	mzTab	jmzTab (Xu, et al., 2014)
Quantitation File Format	mzTab	jmzTab (Xu, et al., 2014)

Table 1: List of supported file formats

1.2.1 Data Model

The Data Object Model comprises a set of Java classes to model the most relevant information included in a MS proteomics experiment, going from the sample preparation to the final experimental results (identification and quantification). All the classes in the Data Object Model extend the *ParamGroup* class to store and handle the associated metadata, e.g. protein scores related terms are stored as controlled vocabulary (CV) terms in the *ParamGroup* class.

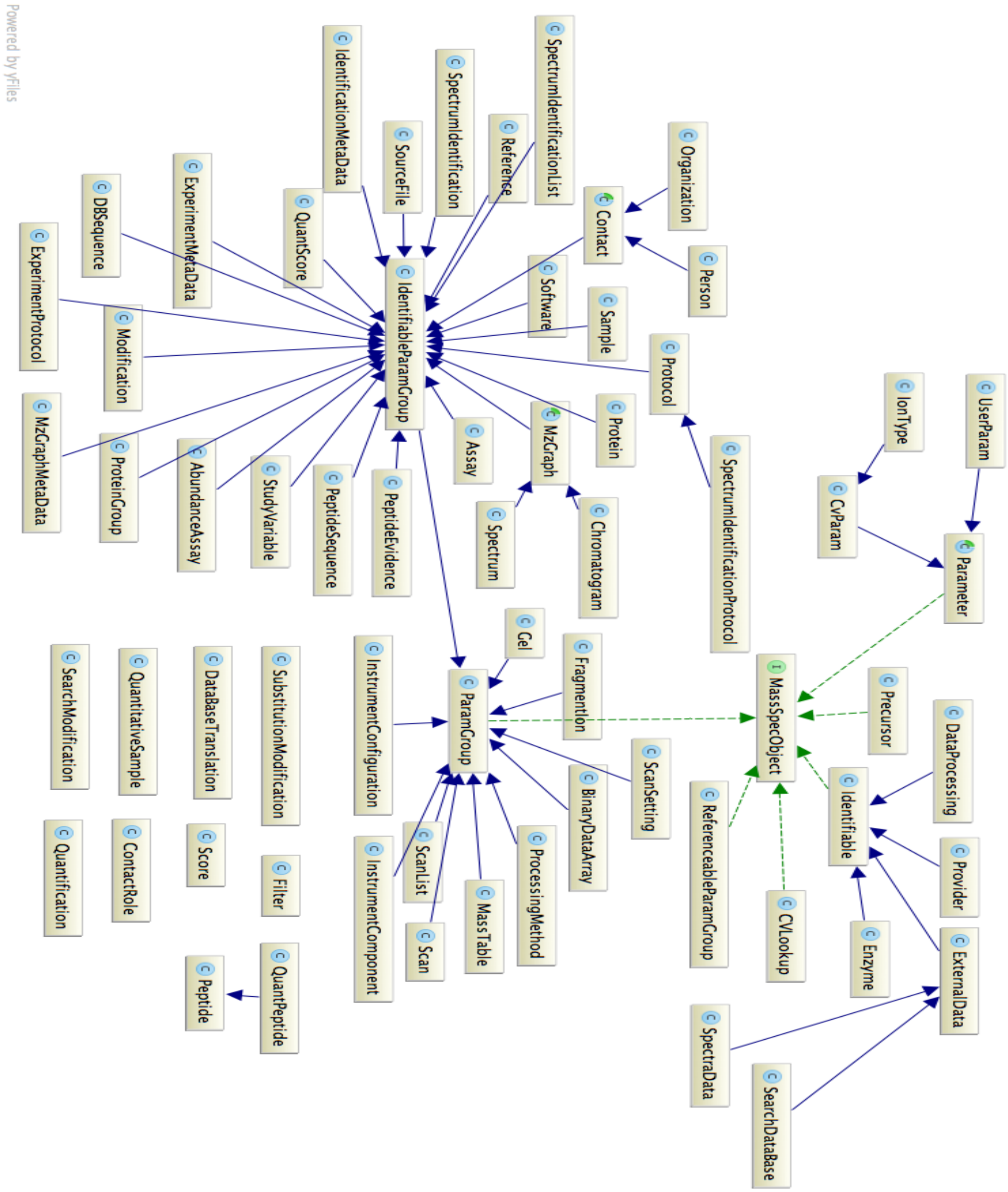

Figure 2: Data Object Model class diagram.

1.2.2 Cache Design

The general idea of caching is to reuse content to avoid repeating expensive operations. In the ms-data-core-api, this is a clear requirement since the average sizes of the experimental output files in proteomics experiments keep increasing. Therefore, loading the entire file in memory is no longer feasible in many cases. On the other hand, requesting the data content directly from the source file is often restricted by the file's storage media. The processing cost for selecting a value from a file (e.g. spectrum, peptide or protein) is fairly high when compared to the cost of having the value stored in memory. Therefore it is plausible to implement caching strategies that keeps frequently used values in the application instead of retrieving these values from the storage media every time. Most frameworks and tools have integrated caching mechanisms nowadays.

Latency and hit rate are the two primary factors to consider when designing a cache. We aimed at achieving a balance between the two while designing the cache for the ms-data-core-api. Therefore, we offer two levels of caching. For *ad-hoc* user interactions (such as the selection of a protein or a peptide), we use the Least Recently Used (LRU) caching algorithm. The main argument behind this algorithm is that when users select a protein/peptide, they are likely to do further investigation on the selected entity. For complex interactions (such as the generation of a file wide plot), another level of caching is designed to enabling the caching of the references between entities and their file system offset. These caching entries will be always available to avoid a full file scan.

While designing cache strategies, one also needs to balance between memory consumption and performance. A critical factor when using caching in Java is the size of the cache: when the cache grows too large, the Java *Garbage Collector* has to clean-up more often (which consumes time). This can lead to a gradual degradation of the performance, or the application may even crash if it exceeds the memory limit.

The ms-data-core-api controls the balance between the memory consumption and fast access to the data using two-level *HashMaps* (Figure 3). Most of the objects such as spectrum, peptide, and protein can be kept in memory for fast access. For this goal, most of the data structures in the API have a key-value representation. A global map is then used to group all the cache structures. The memory defines a cache size for each structure in order to avoid out-of-memory problems. Some values such as precursor ion mass and precursor ion charge are also stored in the cache since these values can frequently be accessed by third-party tools.

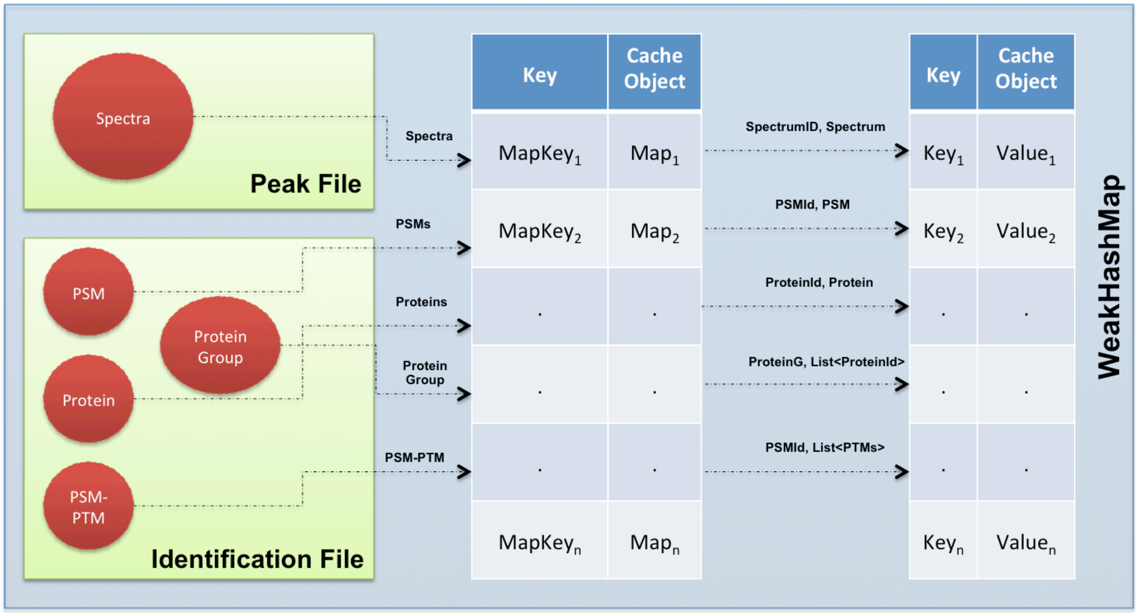

Figure 3: The design of the cache layer.

In the cache maps, not only complete objects are stored, the relationships between some frequently accessed data structures and their properties are also saved. This is one key feature of the library: it allows fast access to the data without the need to load the complete data structure in memory. For example, the cache modification map ensures fast access to the modifications of each PSM without the need to retrieve all the PSMs from the identification file.

The ms-data-core-api depends on a series of libraries and native readers developed by the PRIDE team and other members of the PSI community. Some of these libraries were improved in terms of functionality and performance. Specifically, the libraries for the XML-based formats such as jmzML (Cote, et al., 2010) and jmzIdentML (Reisinger, et al., 2012) were optimized in terms of performance enabling for the first time to load large mzIdentML files with the corresponding mass spectra files (Table 2).

% Decrease loading time compared with previous version	mzIdentML File size (Mb)
30%	4.62
25%	10.10
3%	21.40
5%	99.00
7%	228.00
1%	228.00
58%	445.00
23%	529.00

Table 2: Performance benchmark of the new implementation of jmzIdentML library (Reisinger, et al., 2012). The benchmark was run using different mzIdentML files during the submission process to PRIDE Archive.

1.2.3 PRIDE Utilities: Computational proteomics functionalities

One of the dependencies of ms-data-core-api is the PRIDE Utilities library (https://github.com/PRIDE-Utilities/pride-utilities). The PRIDE Utilities module contains a series of algorithms that extend the functionality of the ms-data-core-api. The definition of the amino acid mass table, *pK* values, and hydrophobic indexes are some of the values defined in PRIDE Utilities. The module also contains the mappings between different controlled vocabulary (CV) or ontology terms meaning the same concept, e.g. the 'b ion' annotation could be annotated using the PRIDE ontology term 'PRIDE:0000194' or the PSI-MS CV term 'MS:1001224'. Therefore, these modules homogenize all the terms and concepts used in metadata annotations. For instance, the library contains the definition of the well-established search engines and processing software. Also, it contains Java-based functions for string validation and complex math functions.

Isoelectric point algorithm: The value of the isoelectric point can be used as a filtering technique to validate peptide identifications (Perez-Riverol, et al., 2012). In the PRIDE Utilities library, the theoretical isoelectric point for proteins and peptides is calculated using a novel method, published by Bjellqvist and coworkers (Bjellqvist, et al., 1993). The *pI* is calculated using *pK* values of the amino acids. These values were defined by examining polypeptide migration between pH 4.5 to 7.3 in an immobilized pH gradient gel environment with 9.2 M and 9.8 M urea. The authors reported a standard deviation of 0.2 units for the entire pH range. A comparison of the algorithm shows that it works for all the fractions and a wide range of pH (Perez-Riverol, et al., 2012). In the future some other implementations could be plugged in the library (Perez-Riverol, et al., 2012).

Gravy Index algorithm: The GRAVY (Grand Average of Hydropathy) value for a peptide or protein is calculated as the sum of hydropathy values (Kyte and Doolittle, 1982) of all the amino acids, divided by the number of residues in the sequence. The gravy index can be used to measure the hydrophobicity of a specific peptide/protein in the sample (Ramos, et al., 2011; Ramos, et al., 2008). The values used for the different amino acids are the following ones:

Amino acid	Value
Ala	1.800
Arg	-4.500
Asn	-3.500
Asp	-3.500

Cys	2.500
Gln	-3.500
Glu	-3.500
Gly	-0.400
His	-3.200
Ile	4.500
Leu	3.800
Lys	-3.900
Met	1.900
Phe	2.800
Pro	-1.600
Ser	-0.800
Thr	-0.700
Trp	-0.900
Tyr	-1.300
Val	4.200

Peptide/Protein Mass: The Peptide/Protein Mass value is computed using each amino acid and the corresponding protein modifications. It is used to compute the delta mass for every peptide sequence and PSM. The mass table used is the following one:

.

Amino acid	Mono Isotopic	Average
Ala	71.03711	71.0788
Arg	156.10111	156.1875
Asn	114.04293	114.1038
Asp	115.02694	115.0886
Cys	103.00919	103.1388

**Gln**	128.05858	128.1307
**Glu**	129.04259	129.1155
**Gly**	57.02146	57.0519
**His**	137.05891	137.1411
**Ile**	113.08406	113.1594
**Leu**	113.08406	113.1594
**Lys**	128.09496	128.1741
**Met**	131.04049	131.1926
**Phe**	147.06841	147.1766
**Pro**	97.05276	97.1167
**Ser**	87.03203	87.0782
**Thr**	101.04768	101.1051
**Trp**	186.07931	186.2132
**Tyr**	163.06333	163.1760
**Val**	99.06841	99.1326

1.2.4 Exporting to mzTab

The ms-data-core-api library includes a set of exporting options from PRIDE XML and mzIdentML files to the mzTab format. The exporting options allow the annotation of mzTab files using the mapping terms from the *pride-utilities* library and annotate the missing metadata in the original files with default information (such as searched database, software and protein modifications terms).

The present version also includes a set of filters to select the high-quality data from mzIdentML files. These filters can be applied when the user is interested in high-quality peptide and protein identifications and not in including all the complete results available in mzIdentML files in the export to mzTab. The current version of the filter includes the following rules:

- If there is not a “protein detection protocol” element in mzIdentML (e.g. no protein ambiguity groups are provided) then the filtering process cannot be done at the protein level directly. In this case:

    - If there is no threshold available at the spectrum identification protocol-> The spectra are filtered using the rank information. Only spectra with rank=1 pass the filter.
    - If there is a threshold available at the spectrum identification protocol-> The spectra are filtered using the provided threshold information.

  Only the proteins whose PSMs remain after the filtering will be kept in the exported mzTab file.

- If there is a "protein detection protocol" element in mzIdentML, the proteins and protein groups will be filtered according to the threshold information first.
  - After that the filtering by threshold at the peptide level will be applied, because in the worst-case scenario it will remove only proteins without spectra evidence that pass the filter. Before, "NoPeptideFilter" was used to avoid inconsistencies with the protein filter. However, it was observed that some spectra evidences that did not pass the threshold were included because the threshold was provided but was incorrectly annotated in the file as "NoThresholdAvailable". This option minimizes the inclusion of spectra that do not pass the threshold.
- If there is no threshold information at the protein or peptide level available:
  - The spectra are filtered using the PSM rank information. Only PSMs with rank=1 pass the filter.
  - Only the proteins whose PSMs remain after the filtering process will be kept in the exported mzTab file.

2. Updated ms-data-core-api dependencies

2.1 PRIDE XML JAXB

PRIDE XML JAXB	
**Website & Source Code**	http://code.google.com/p/pride-toolsuite/wiki/PRIDEXMLJAXB
**Description**	PRIDE XML JAXB library is a library for indexing and parsing PRIDE XML 2.1 [http://www.ebi.ac.uk/pride/schemaDocumentation.do] files. Unlike the conventional way of loading XML files, this library **does not** load the whole file into the memory up-front, instead it an employs XML indexing technique to index the file on the fly which results in fast access and a small memory footprint. Additionally, all entities from a PRIDE XML file are mapped into objects, and the internal references between the objects are resolved automatically. This gives direct access in the object model to entities that are only referenced by ID in the actual XML file.
**License**	Apache 2 open source license
**Language**	Java
**External libraries**	XXIndex [http://code.google.com/p/pride-toolsuite/wiki/XXIndex]: Indexing XML files.
	JAXB [http://jaxb.java.net/]: Parsing XML snippets into object model.

2.2 jmzML

jmzML	
**Website & Source Code**	http://code.google.com/p/jmzml/
**Description**	jmzML provides a portable and lightweight JAXB-based implementation of the full mzML 1.1 standard format (http://www.psidev.info/mzml). mzML files are effectively indexed on the fly and used as swap files, with only requested snippets of data loaded from a file when accessing it. Additionally, internal references in the mzML XML can be resolved automatically by jmzML, giving direct access in the object model to entities that are only referenced by ID in the actual XML file. Apart from reading indexed and non-indexed

	mzML files, jmzML also enables the writing of non-indexed mzML files.
**License**	Apache 2 open source license
**Language**	Java
**External libraries**	XXIndex [http://code.google.com/p/pride-toolsuite/wiki/XXIndex]: Indexing XML files.
	JAXB [http://jaxb.java.net/]: Parsing XML snippets into object model.

2.3 jmzIdentML

**jmzIdentML**	
**Website & Source Code**	https://code.google.com/p/jmzidentml/
**Description**	A Java API to the Proteomics Standards Initiative's mzIdentML format (version 1.1 - PSI stable version). The mzIdentML data standard captures peptide and protein identification data, generated from mass spectrometry. For more information about mzIdentML, see the relevant googlecode repository: http://code.google.com/p/psi-pi/. The API can be imported directly as a jar file to provide access to read and write functionality for mzIdentML. The API builds on top of JAXB capabilities, by providing an indexing scheme that allows random access to parts of the file. Under the wiki there are some code snippets showing example usage.
**License**	Apache 2 open source license
**Language**	Java
**External libraries**	XXIndex [http://code.google.com/p/pride-toolsuite/wiki/XXIndex]: Indexing XML files.
	JAXB [http://jaxb.java.net/]: Parsing XML snippets into object model.

2.4 jmzReader

<table>
<tr><th colspan="2">jmzReader</th></tr>
<tr><td>Website & Source Code</td><td>https://code.google.com/p/jmzreader/</td></tr>
<tr><td>Description</td><td>The jmzReader library is a collection of Java APIs to parse the most commonly used MS peak list formats. Currently, the library contains parsers for:
• dta
• mgf
• ms2
• mzData
• mzXML
• pkl
• mzML
• apl

All parsers are optimized to be used in conjunction with mzIdentML (see link in the left panel). Based on a custom build class to efficiently parse text files line by line all parsers can handle arbitrary large files in minimal memory, allowing easy and efficient processing of peak list files using the Java programming language. mzIdentML files do not contain spectra data but refer to external peak list files. All peak list parsers support the methods used by mzIdentML to reference external spectra and implement a common interface. Thus, when developing software for mzIdentML programmers no longer have to support multiple peak list file formats but only this one interface.</td></tr>
<tr><td>License</td><td>Apache 2 open source license</td></tr>
<tr><td>Language</td><td>Java</td></tr>
<tr><td rowspan="2">External libraries</td><td>XXIndex [http://code.google.com/p/pride-toolsuite/wiki/XXIndex]: Indexing XML files.</td></tr>
<tr><td>JAXB [http://jaxb.java.net/]: Parsing XML snippets into object model.</td></tr>
</table>

3. Algorithms and tools built on top of the ms-data-core-api

A set of different algorithms and tools has been developed on top of the ms-data-core-api. In this section we will describe some of these libraries and tools.

3.1 PRIDE Inspector Toolsuite

The new version of PRIDE Inspector tool (https://github.com/PRIDE-Toolsuite/pride-inspector) makes use of the ms-data-core-api as the main library for data source handling and representation. The main goal of this tool is to visualize the ProteomeXchange 'complete' submissions in PRIDE (Figure 4).

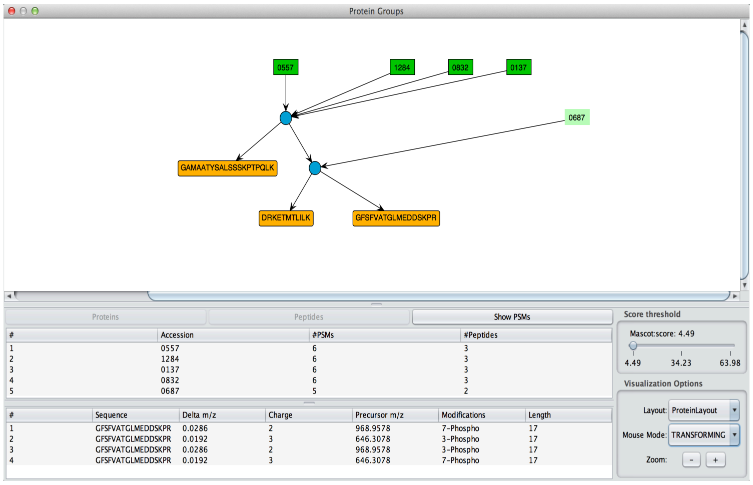

Figure 4: Protein group visualization panel in PRIDE Inspector (https://github.com/PRIDE-Toolsuite/inspector-example-files/tree/master/mzIdentML/MS-GF+)

The new PRIDE Inspector 2 supports the visualization of spectra, chromatograms, protein groups, proteins, PSMs and the corresponding metadata (scores, thresholds, quantitative values, etc).

3.2 PRIDE submission pipeline

The PRIDE database (http://www.ebi.ac.uk/pride/archive/) (Vizcaino, et al., 2013) makes use of the ms-data-core-api during the submission process of ProteomeXchange 'complete' submissions. The 'complete' submissions are based on the file formats mzIdentML and PRIDE XML. Additionally, they also contain a set of mass spectra files associated with the identification files that are handled during the submission process. All the properties related with each assay such as samples details, instruments, number of identified proteins, number of unique peptides, among others, are retrieved from the files using the ms-data-core-api (Figure 5).

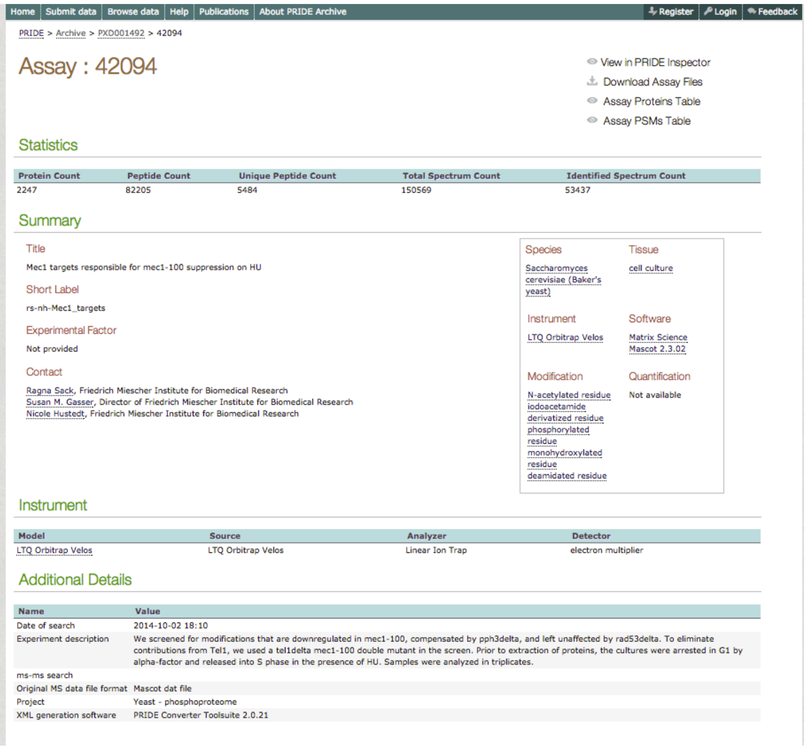

Figure 5: Assay view in the PRIDE Archive web showing the information coming from a PRIDE XML file (number of proteins, peptides, unique peptides, total number of spectra, etc).

3.3 HI-bone

HI-bone (Perez-Riverol, et al., 2013) is an approach for scoring MS/MS identifications based on the high mass accuracy matching of precursor ions, the identification of a high intensity b1 fragment ion, and partial sequence tags from phenylthiocarbamoyl-derivatized peptides. This derivatization process boosts the b1 fragment ion signal, which turns it into a powerful feature for peptide identification. The ms-data-core-api is used to retrieve the information of each spectrum and to store the results.

3.4 Protein Inference Algorithms (PIA)

The Protein Inference Algorithm (PIA) suite (https://github.com/mpc-bioinformatics/pia) written in Java, includes a fully parametrisable web-interface (using Java Server Faces), which combines PSMs from different experiments and/or search engines, and reports consistent and comparable results (Figure 6). None of the parameters for the protein inference process (e.g. filtering or scoring), are fixed like in prior approaches. Instead they are held as flexible as possible, to enable any adjustments needed by the user.

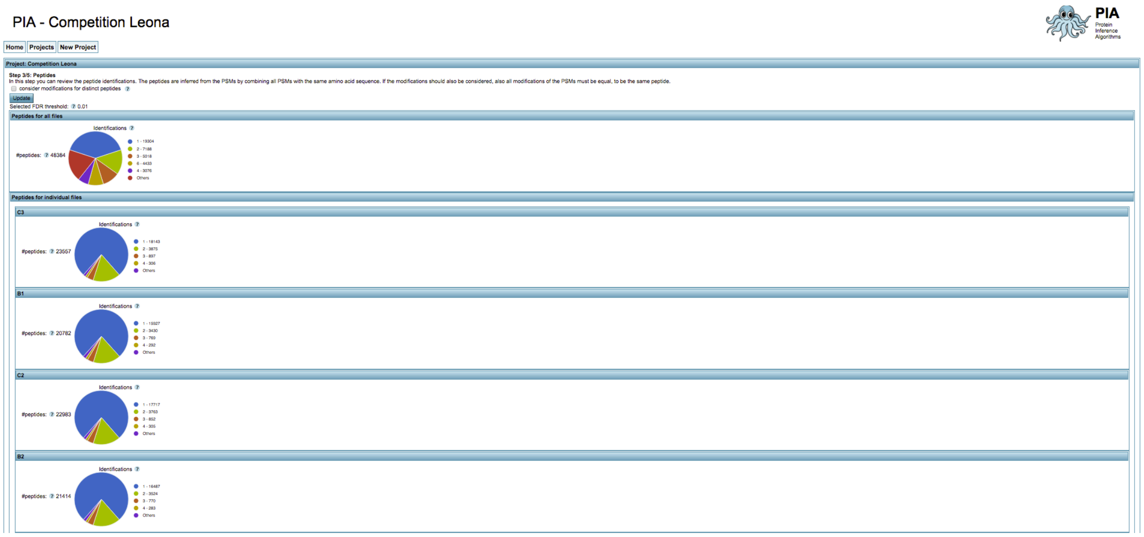

Figure 6: PIA web interface.

The library was built on top of the ms-data-core-api demonstrating that the developers only need to focus on the new algorithms and tools avoiding details related with the common data structures and file handling. The PIA set of algorithms can be applied to all the ms-data-core-api supported formats containing peptide identification data (PRIDE XML, mzIdentML and mzTab).

4. Coding examples

A list of examples can be found in the GitHub page of the project (https://github.com/PRIDE-Utilities/ms-data-core-api).

4.1 Read an mzXML file

```
-----------------------------------------------------------------------------------------

//Reading an mzXML file
DataAccessController mzXmlController = new MzXmlControllerImpl(new File(filename.mzXML));
List<Comparable> ids = new ArrayList<Comparable>(mzXmlController.getSpectrumIds());
assertTrue("The id of the first spectra should be", ids.contains("1"));

//Return the first spectrum from an mzXML file
Spectrum spectrum = mzXmlController.getSpectrumById(ids.get(0));


------------------------------------------------------------------------------------------
```

4.2 Read an mzIdentML file

```
------------------------------------------------------------------------------------------

//Reading a mzIdentML file
mzIdentMlController = new MzIdentMLControllerImpl(new File("identification.mzid"));
List<Sample> samples = mzIdentMlController.getSamples();

//Checking the contact details
List<Person> persons = mzIdentMlController.getPersonContacts();
------------------------------------------------------------------------------------------
```

4.3 Retrieve the isoelectric point of all the identified peptides

```
------------------------------------------------------------------------------------------

//Reading a mzIdentML file
mzIdentMlController = new MzIdentMLControllerImpl(new File("identification.mzid"));


//Retrieve all Protein Ids
```

```
Collection<Comparable> proteinIds = mzIdentMlController.getProteinIds();
for(Comparable proteinID: proteinIds){
        Collection<Comparable> peptideIds = mzIdentMlController.getPeptideIds(proteinID);
        //Retrieve for every Protein the corresponding Peptides
        for(Comparable peptideId: peptideIds){
                //Compute the isoelectric point
                System.out.println(IsoelectricPointUtils.calculate(mzIdentMlController.getPeptideSequence(
        proteinID,peptideId)));
        }
}
------------------------------------------------------------------------------------------
```

4.4 Export PRIDE XML to mzTab

```
------------------------------------------------------------------------------------------
//Reading a mzIdentML file
controller = new PRIDEControllerImpl(new File("identification.xml"));
// Create the exporter
AbstractMzTabConverter mzTabconverter = new PRIDETabConverter(controller);

//Create a checker to analyze the exported file
MZTabFile mzTabFile = mzTabconverter.getMZTabFile();
MZTabFileConverter checker = new MZTabFileConverter();
checker.check(mzTabFile);

//Export to an output file
mzTabFile.printMZTab(new FileOutputStream("output.mztab"));


------------------------------------------------------------------------------------------
```

4.5 Export mzIdentML information and protein inference to mzTab

```
controller = new MzIdentMLControllerImpl(new File("identification.mzid"));
PIAModeller piaModeller = new PIAModeller();
CvScore cvScore = null;
String scoreAccession = null;
// try to get the main-score
for (SearchEngineScoreCvTermReference termRef : controller.getAvailablePeptideLevelScores()) {
    CvScore newCvScore;
    scoreAccession = termRef.getAccession();
    newCvScore = CvScore.getCvRefByAccession(termRef.getAccession());
      if ((newCvScore != null) && newCvScore.getIsMainScore()) {
           cvScore = newCvScore;
           scoreAccession = cvScore.getAccession();
```

```
            break;
        }
}

// add the input file to modeller and import data
Integer controllerID = piaModeller.addPrideControllerAsInput(controller);
piaModeller.importAllDataFromFile(controllerID);

// first create the intermediate structure from the data given by the controller

piaModeller.buildIntermediateStructure();
PeptideScoring pepScoring = new PeptideScoringUseBestPSM(scoreAccession, false);
ProteinScoring protScoring;
if ((cvScore != null) && !cvScore.getHigherScoreBetter()) {
  protScoring = new ProteinScoringMultiplicative(false, pepScoring);
} else {
  protScoring = new ProteinScoringAdditive(false, pepScoring);
}

// perform the protein inferences
piaModeller.getProteinModeller().infereProteins(pepScoring, protScoring, OccamsRazorInference.class,
null, false);

// create the protein groups
int nrGroups = piaModeller.getProteinModeller().getInferredProteins().size();
Map<Comparable, Map<Comparable, List<Comparable>>> prideProteinGroupMapping = new HashMap<Comparable,
Map<Comparable,List<Comparable>>>(nrGroups);

for (InferenceProteinGroup piaGroup : piaModeller.getProteinModeller().getInferredProteins()) {
    Map<Comparable, List<Comparable>> proteinPeptideMap = null;
    Set<IntermediateProtein> proteinSet = new HashSet<IntermediateProtein>(piaGroup.getProteins());
    // include the subGroups
    for (InferenceProteinGroup subGroup : piaGroup.getSubGroups()) {
         proteinSet.addAll(subGroup.getProteins());
    }

    proteinPeptideMap = new HashMap<Comparable, List<Comparable>>(proteinSet.size());
    for (IntermediateProtein protein : proteinSet) {
        Comparable proteinID = ((PrideIntermediateProtein)protein).getPrideProteinID();
        proteinPeptideMap.put(proteinID, null);
    }
    prideProteinGroupMapping.put(piaGroup.getID(), proteinPeptideMap);
}
//Set the protein groups and inference information in the current model.
controller.setInferredProteinGroups(prideProteinGroupMapping);
//Export to mzTab
AbstractMzTabConverter mzTabconverter = new MzIdentMLMzTabConverter(controller);
MZTabFile mzTabFile = mzTabconverter.getMZTabFile();
MZTabFileConverter checker = new MZTabFileConverter();
checker.check(mzTabFile);
//Export to an output file
mzTabFile.printMZTab(new FileOutputStream("output.mztab"));
```

5. References

Bjellqvist, B., *et al.* The focusing positions of polypeptides in immobilized pH gradients can be predicted from their amino acid sequences. *Electrophoresis* 1993;14(10):1023-1031.

Cote, R.G., Reisinger, F. and Martens, L. jmzML, an open-source Java API for mzML, the PSI standard for MS data. *Proteomics* 2010;10(7):1332-1335.

Griss, J., *et al.* The mzTab data exchange format: communicating mass-spectrometry-based proteomics and metabolomics experimental results to a wider audience. *Molecular & cellular proteomics : MCP* 2014;13(10):2765-2775.

Griss, J., *et al.* jmzReader: A Java parser library to process and visualize multiple text and XML-based mass spectrometry data formats. *Proteomics* 2012;12(6):795-798.

Jones, A.R., *et al.* The mzIdentML data standard for mass spectrometry-based proteomics results. *Molecular & cellular proteomics : MCP* 2012;11(7):M111 014381.

Kyte, J. and Doolittle, R.F. A simple method for displaying the hydropathic character of a protein. *Journal of molecular biology* 1982;157(1):105-132.

Martens, L., *et al.* mzML--a community standard for mass spectrometry data. *Molecular & cellular proteomics : MCP* 2011;10(1):R110 000133.

Perez-Riverol, Y., *et al.* Isoelectric point optimization using peptide descriptors and support vector machines. *Journal of proteomics* 2012;75(7):2269-2274.

Perez-Riverol, Y., *et al.* HI-bone: a scoring system for identifying phenylisothiocyanate-derivatized peptides based on precursor mass and high intensity fragment ions. *Analytical chemistry* 2013;85(7):3515-3520.

Ramos, Y., *et al.* Peptide fractionation by acid pH SDS-free electrophoresis. *Electrophoresis* 2011;32(11):1323-1326.

Ramos, Y., *et al.* Proteomics based on peptide fractionation by SDS-free PAGE. *Journal of proteome research* 2008;7(6):2427-2434.

Reisinger, F., *et al.* jmzIdentML API: A Java interface to the mzIdentML standard for peptide and protein identification data. *Proteomics* 2012;12(6):790-794.

Vizcaino, J.A., *et al.* The PRoteomics IDEntifications (PRIDE) database and associated tools: status in 2013. *Nucleic acids research* 2013;41(Database issue):D1063-1069.

Xu, Q.W., *et al.* jmzTab: a java interface to the mzTab data standard. *Proteomics* 2014;14(11):1328-1332.
